# A New Method for Olive Oil Screening Using Multivariate Analysis of Proton NMR Spectra

**DOI:** 10.3390/molecules27010213

**Published:** 2021-12-30

**Authors:** Colleen L. Ray, James A. Gawenis, C. Michael Greenlief

**Affiliations:** 1 Department of Chemistry, University of Missouri, 601 S. College Avenue, Columbia, MO 65211, USA; clrxtc@mail.missouri.edu; 2 Sweetwater Science Laboratories, Glasgow, MO 65264, USA; jim@sweetwaterscience.com

**Keywords:** proton NMR, food authenticity, adulteration, olive oil, edible oil, PCA

## Abstract

A new NMR-based method for the discrimination of olive oils of any grade from seed oils and mixtures thereof was developed with the aim of allowing the verification of olive oil authenticity. Ten seed oils and seven monovarietal and blended extra virgin olive oils were utilized to develop a principal component analysis (PCA) based analysis of ^1^H NMR spectra to rapidly and accurately determine the authenticity of olive oils. Another twenty-eight olive oils were utilized to test the principal component analysis (PCA) based analysis. Detection of seed oil adulteration levels as low as 5% *v*/*v* has been shown using simple one-dimensional proton spectra obtained using a 400 MHz NMR spectrometer equipped with a room temperature inverse probe. The combination of simple sample preparation, rapid sample analysis, novel processing parameters, and easily interpreted results, makes this method an easily accessible tool for olive oil fraud detection by substitution or dilution compared to other methods already published.

## 1. Introduction

Olive oil is the oil collected from the fruit of the olive tree (*Olea europaea* L.) typically through simple mechanical pressing. Olive oil is somewhat unusual as the oil is extracted from the flesh of the fruit instead of the seed as is common in most other food oils. This oil has been consumed by humans since antiquity and remains a highly valued food oil today. Due to the high market price of olive oil compared to other oils, it is a popular target for adulteration through dilution with other oils or label fraud by selling non-olive sourced oils as genuine olive oil [1]. The aim of this work is to develop a rapid analysis for the detection of seed oil adulteration in any grade of olive oil.

The motivation for adulteration is one of simple greed. If olive oil is diluted with a less costly oil or is completely replaced by said oil, the profits from selling it as genuine olive oil can be quite large. Adulteration of olive oils can affect consumers beyond the obvious economic impact of paying a premium for fraudulent goods. Olive oil is often consumed for its reputed health benefits due to its unique composition, which would be reduced if diluted or absent if the product contains no olive oil whatsoever. Inadvertent consumption of oils ordinarily avoided by people with allergies could have significantly more serious and immediate effects on the consumer if the so-labeled olive oil contains products to which the consumer is sensitive.

NMR spectroscopy has long been the gold standard method for the elucidation of unknown molecular structures and is often used in synthetic chemistry for verification of products. In recent years the ability for NMR spectroscopy to screen products and materials for quality control or authenticity has gained significant attention. The data analysis methods employed in this study are broadly similar to those used for untargeted metabolomic fingerprinting commonly performed with mass spectrometry. While mass spectrometry has greater sensitivity, NMR is capable of more rapidly screening complex mixtures such as foodstuffs in a non-destructive manner. Coupling NMR results with principal component analysis (PCA) allows even subtle differences in overall composition to be useful for discriminating between oil sources and detecting adulterated samples. Utilizing a 400 MHz NMR to detect lower levels of adulteration of olive oil with oils such as high-oleic acid cultivars of sunflower and safflower oil is challenging due to these adulterants having lipid profiles very similar to those found in olive oils [2].

Olive oil is composed of fatty acid triglycerides with lower concentrations of a variety of phenolic and polyphenolic compounds [3]. Oleic acid is the most abundant fatty acid found in olive oils with varying levels of linoleic acid, linolenic acid, and palmitic acid. The ratios of the various fatty acids in olive oil differs from those found in many seed oils, particularly due to the high levels of oleic acid and low levels of ω-3 α-linolenic acid. The spectral signature of the lower concentration fatty acids contributes significantly to differentiating olive oils from high oleic acid varietals of seed oils.

**Figure 1:**
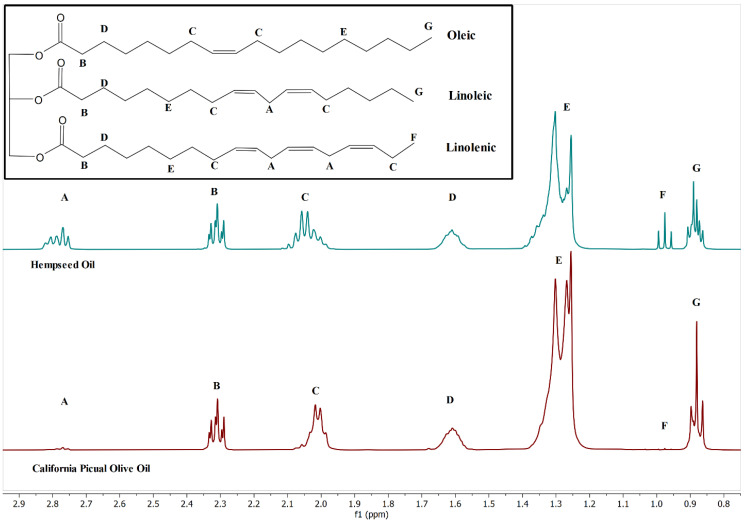
Comparison of 400 MHz ^1^H NMR spectra of hempseed and olive oil with model triglyceride structure detailing origins of specific labeled peaks from various fatty acid subgroups.

These differing levels manifest themselves spectrally and allow these oils to be differentiated via analysis. A spectral comparison of hempseed oil and a sample of monovarietal picual olive oil demonstrates many of these differences [4] (Figure 1). The triplet “F” at 0.977 ppm arises from the terminal methyl group of ω-3 fatty acids, most often α-linolenic acid, which is found in significantly higher concentrations in hempseed oil than olive oil. The proximity of the π-bond between carbons 2 and 3 in ω-3 fatty acids deshields the terminal methyl group resulting in a shift away from peak “G” at 0.881 ppm belonging to the same functional groups in other fatty acids.

Multiplet “A” at 2.78 ppm (Figure 1) is due to the methylene protons positioned between two π-bonds in polyunsaturated fatty acids, such as linoleic and linolenic. Polyunsaturated fatty acids are plentiful in hempseed oil and are in relatively low abundance in olive oils as seen by the intensity difference of this feature in the two spectra [4].

Analyzing these spectra manually via peak area ratiometrics is possible but is time intensive and tedious due to the number of peaks and variables involved. Utilizing PCA to group similar spectra together accomplishes a similar overall goal while being far easier to automate and produces easily interpreted results [5]. PCA is often used in many complex analyses and has been shown here to work well to discriminate between spectra of various food oils. Not only is the method described herein able to differentiate pure oils by source, but the method can also detect olive oil that has been diluted by other oils.

Olive oil authenticity testing via gas chromatography (GC) and liquid chromatography (LC) are well established methods. These GC and LC methods are comparatively slow, often involving several sample preparation steps, and sample analysis runs on the order of 30 min with longer run times being commonplace [6,7]. NMR analysis of oil samples requires no sample preparation aside from mixing the sample with a deuterated solvent and experiment run times are on the order of 15 min for a simple one-dimensional proton NMR. PCA of food oil NMR spectra is not entirely a novel development in and of itself [8,9]. However, previous studies did not demonstrate the ability to detect adulteration via dilution, and generally dealt with differentiating olive oils by geographical location. These studies also used NMR spectrometers with higher field magnets that are more expensive and less widely available than the comparatively inexpensive 400 MHz system used in this study.

## 2. Materials and Methods

### 2.1. Chemicals and Materials

Deuterated chloroform (CDCl_3_ 99.9% D, 1% *w*/*w* TMS) was obtained from Acros Organics (Fair Lawn, NJ, USA).

### 2.2. Oil Samples

Olive oil and seed oil samples were purchased from local and online retailers. Olive oil samples consisted of monovarietal and blended oils of European, Mediterranean, and North American origin. A single premixed blend of 70% canola, 20% olive oil, and 10% (all *v*/*v*) grapeseed oil was used as a blended sample for comparison. Table 1 lists the olive oil samples used in this study.

The seed oils used for comparison and adulteration studies were: almond, argan, high-oleic canola, cottonseed, grapeseed, hazelnut, hempseed, peanut, high-oleic safflower, soybean, and high-oleic sunflower oils. All seed oils were purchased from online and local retailers.

### 2.3. Sample Preparation

50 µL of oil was added directly to a clean 5 mm NMR tube (Deutero Boroeco 8, Deutero GmbH, Kastellaun, Germany) with a pipette. 550 µL of CDCl_3_ was then added to the NMR tube. The tube was then capped, inverted to ensure complete mixing, and then analyzed.

#### Adulterated Samples

Sample 20, a coratina monovarietal olive oil from California, was mixed with varying levels of canola, hazelnut, peanut, safflower, and sunflower oils to test the ability of this method to detect adulteration via dilution. Canola and hazelnut oil adulteration samples were prepared with 10%, 15%, and 20% (*v*/*v*) of adulterant. The same olive oil was also adulterated with 10%, 20%, 30%, and 40% peanut, safflower, and sunflower oils. A premixed commercially available blend of 70% canola, 20% olive oil, and 10% grapeseed oil (all *v*/*v*) was also analyzed to further test the model.

All adulterated samples were prepared to a final volume of 5 mL. Olive oil and adulterants were measured into a 15 mL conical tube, vortexed for 20 s to ensure complete mixing, and prepared for analysis as described in Section 2.3.

### 2.4. NMR Analysis

NMR spectra were collected using a Bruker Avance IIIHD spectrometer operating at 400.13 MHz. The probe used was a 5 mm BBI room temperature probe. The sample temperature was 298 K. A simple proton experiment was performed (30° pulse, 64 scans, 2 dummy scans, 20 ppm sweep width, 65,536 data points). A 10 s relaxation delay was used in order to ensure complete relaxation between scans based upon a 1 s T_1_ measurement.

### 2.5. Spectral Processing Parameters

Spectral processing was performed with Mestrenova 14.1 (Mestrelab, Santiago de Compostela, Spain). Spectral processing parameters described in Table 2.

### 2.6. Principal Component Analysis

PCA analysis was performed using Mestrenova 14.2 (Mestrelab, Santiago de Compostela, Spain). The PCA analysis was blinded to six regions to eliminate portions of the spectrum irrelevant to oil analysis using the parameters outlined in Table 3. The PCA settings were as follows: Binning mode regular with summed intensity, bin width: 0.05 ppm. Pareto scaling was applied.

## 3. Results

### 3.1. Normalization of Spectra

**Figure 2:**
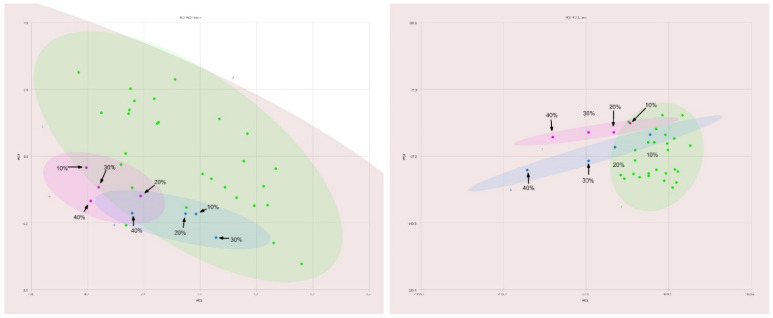
Comparison of PC1–PC2 plots of 400 MHz 1H NMR spectra of pure olive oils (green), safflower oil adulterated olive oil (magenta) and sunflower oil adulterated olive oil (blue) showing the differences between traditional normalization (**left**) and normalization to ω-3 fatty acids (**right**).

As shown in Figure 2, reasonable grouping of olive oils (green) was observed with principal components 1 and 2 accounting for 97.7% and 1.8% of total variance, respectively. The blue- and magenta-colored ellipses contain information about the adulterated olive oil samples. The percentages shown on the figure indicate the amount of adulterant oil added to olive oil. In the PC1-PC2 plot on the left-hand side, the intensities are normalized to the tallest peak in the spectrum. It is not possible to differentiate these samples easily even at adulteration levels of 40% *v*/*v*. However, when the spectra are normalized to the ω-3 methyl signal, as shown in right hand side of Figure 2, this technique becomes significantly more sensitive to adulteration with these oils and also shows far tighter grouping of olive oil samples. Based on these results, all spectra were normalized to the ω-3 methyl signal in the remainder of the study.

### 3.2. Differentiation of Olive and Seed Oils

**Figure 3:**
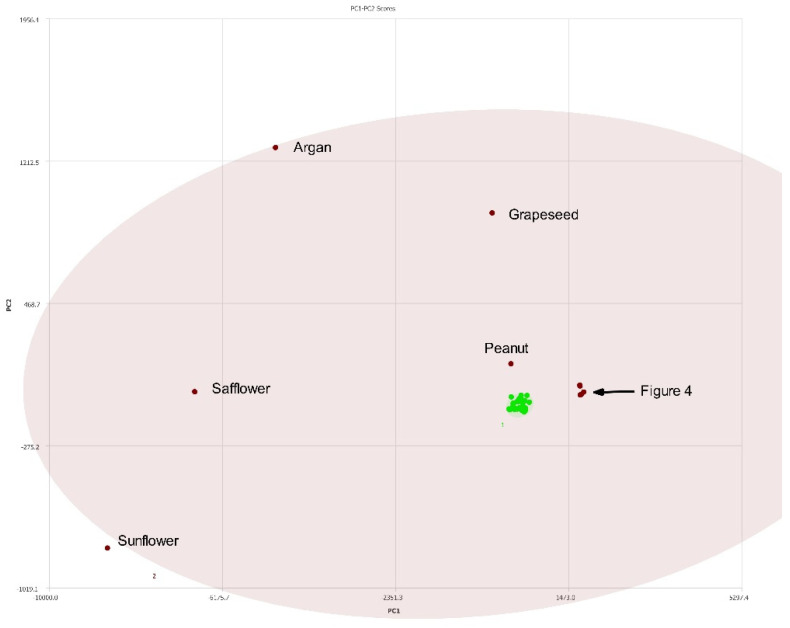
PC1–PC2 plot of 400 MHz ^1^H NMR spectra of seed oils (labeled) versus olive oils (green). Ellipses represent the 95% confidence interval. The congested region is expanded in Figure 4.

**Figure 4:**
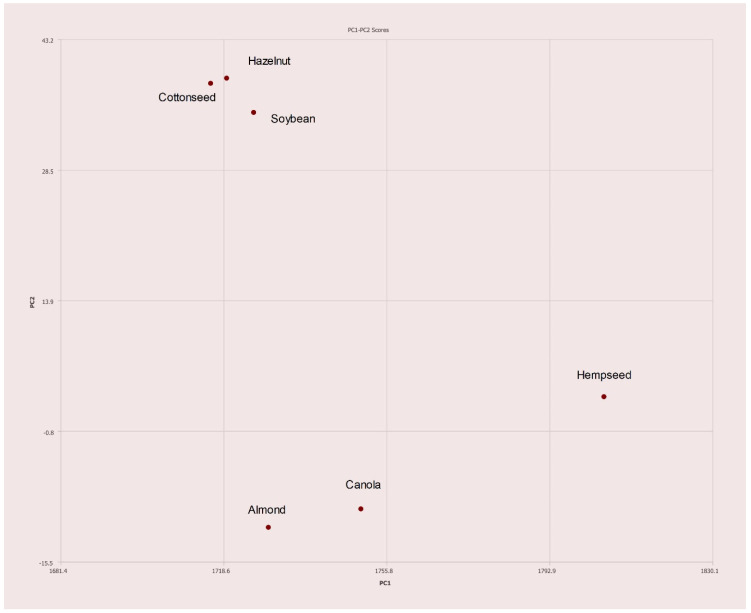
PC1–PC2 plot expansion of congested region in Figure 3.

A total of 28 single varietal and blended olive oils and 10 seed oils were analyzed via NMR with PCA performed on the collected spectra. Figure 3 summarizes the results. The cluster of green dots are the different olive oils samples and are observed in a tight cluster. The seed oils are shown as maroon dots and are clearly separated from the olive oils. There is a cluster of seed oils (maroon) in Figure 3 to the right of the olive oils. This region is expanded in Figure 4. The expanded region clearly shows the differences between hazelnut oil and hempseed oil, for example.

### 3.3. Testing against Mixtures of Olive Oil and Seed Oils

**Figure 5:**
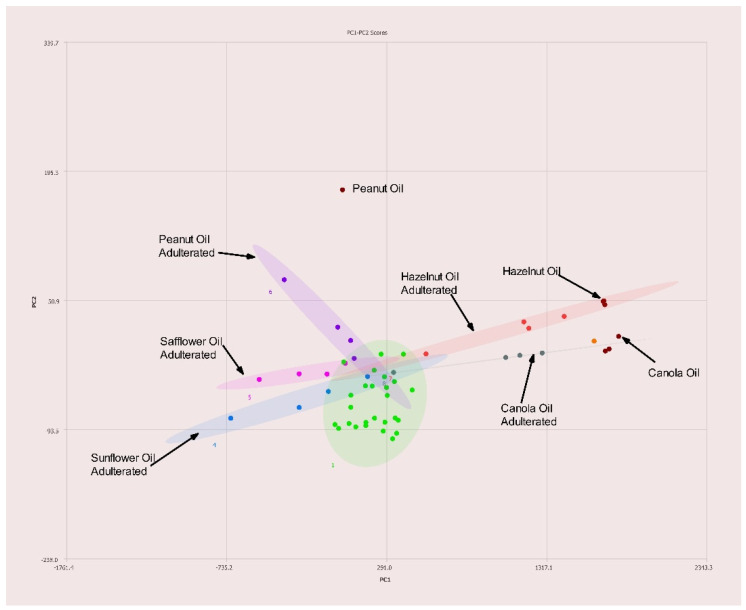
PC1–PC2 plot of 400 MHz ^1^H NMR spectra of seed oils (labeled) versus olive oils (green). Ellipses represent the 95% confidence interval.

In Figure 5, the green dots represent the same olive oils shown in Figure 2. The extended ellipses represent olive oils samples that have been adulterated as described in the previous section. To the far right of the figure, the labeled maroon dots indicate the location of pure hazelnut oil (also indicated by an arrow in the figure). As the concentration of olive oil is increased the red dots show how the samples moved towards the pure olive oil region (green). Four other oils were used to dilute olive oil. In each case, the undiluted sample (pure olive oil) resides in the green region. As the concentration of the adulterant oil increases, the ellipses move towards the respective oil used to dilute the olive oil (peanut, safflower, sunflower, or canola oil). Due to the greater degree of separation, particularly in the case of high-oleic canola, safflower, and sunflower oils, this allows for identification of the adulterant oil.

**Figure 6:**
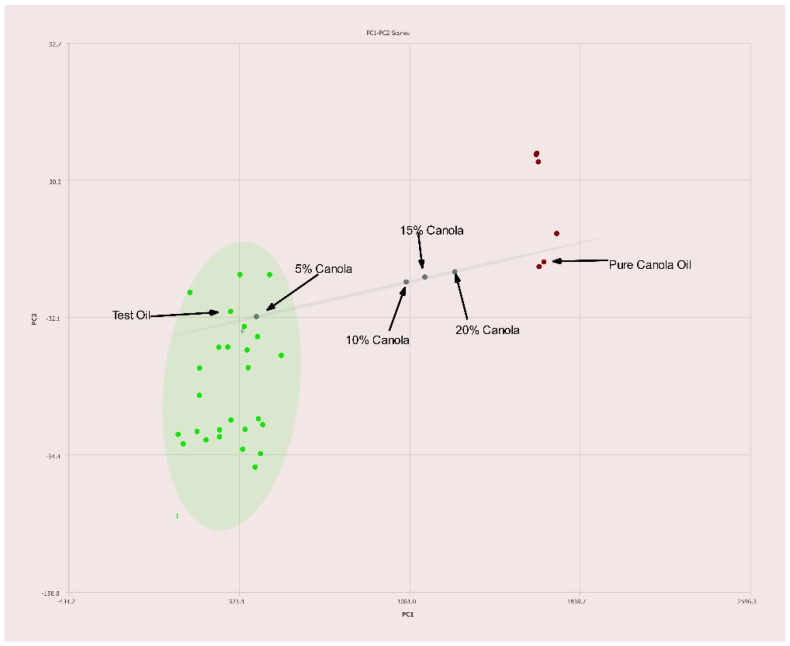
PC1–PC2 plot of 400 MHz ^1^H NMR spectra of olive oil adulterated with canola oil with percentages of adulteration noted. Ellipses represent the 95% confidence interval.

Figure 6 shows the results for different concentrations of canola oil compared to different olive oils. Pure canola oil shows excellent separation from pure olive oils as shown in Figure 6 with an ability to discriminate pure olive oils from those adulterated with canola at levels under 10% *v*/*v*.

**Figure 7:**
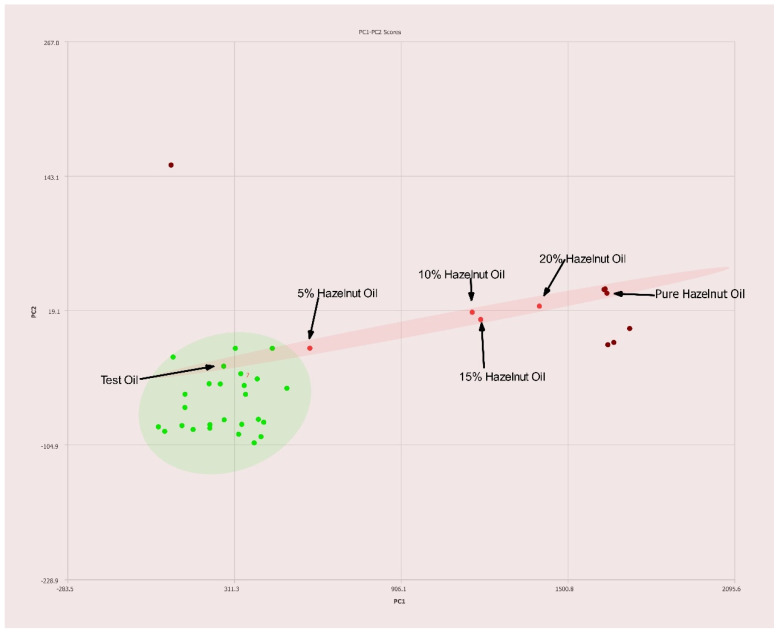
PC1–PC2 plot of 400 MHz ^1^H NMR spectra of olive oil adulterated with hazelnut oil with percentages of adulteration noted. Ellipses represent the 95% confidence interval.

The results from the same type of experiment, but using hazelnut oil adulterated olive oil is easily detected even at levels as low as 5% *v*/*v* are shown in Figure 7. The pure hazelnut oil is easily distinguished from pure olive oil. The adulterated samples following a grouping between the two pure oils as the concentration of adulterated hazelnut oil is varied.

**Figure 8:**
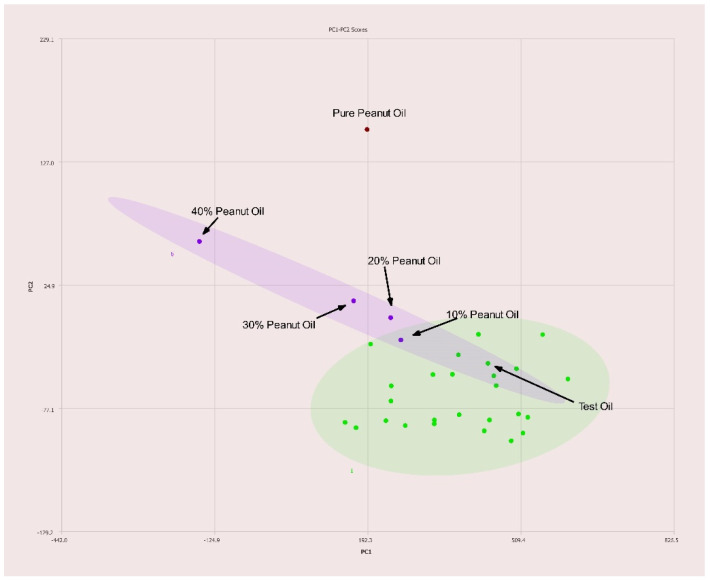
PC1–PC2 plot of 400 MHz ^1^H NMR spectra of olive oil adulterated with peanut oil with percentages of adulteration noted. Ellipses represent the 95% confidence interval.

Peanut oil adulterated olive oil is detectable at concentrations below 20% *v*/*v* as shown in Figure 8. Again, pure peanut oil is easily separated from pure olive oil samples using the PCA analysis.

**Figure 9:**
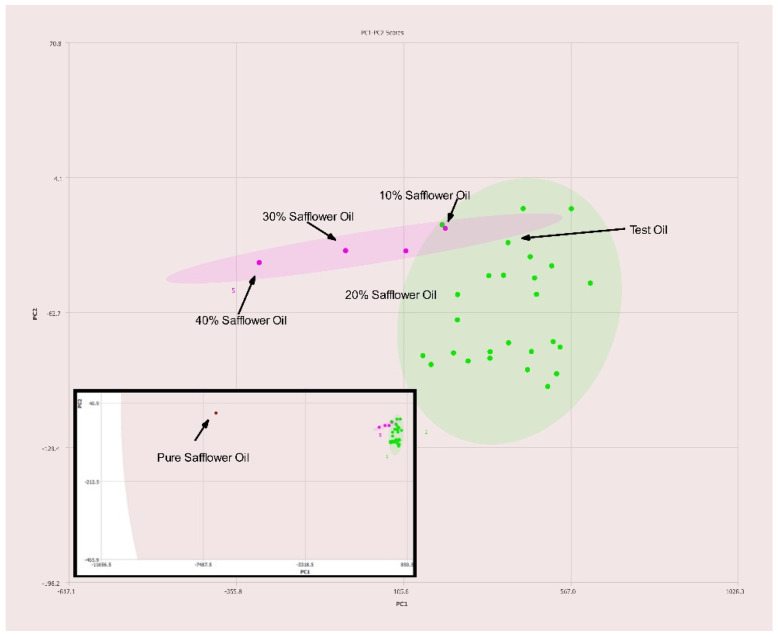
PC1–PC2 plot of 400 MHz ^1^H NMR spectra of olive oil adulterated with safflower oil with percentages of adulteration noted. Ellipses represent the 95% confidence interval. The inset shows the expanded PCA plot to show the position of pure safflower oil with respect to the pure olive oil samples.

High oleic acid safflower oil is ordinarily rather difficult to distinguish from olive oils due to having similar lipid profiles. However, using this method it is detectable in levels slightly under 20% *v*/*v* as observed in Figure 9. Samples above this level are readily detected as not being olive oil.

**Figure 10:**
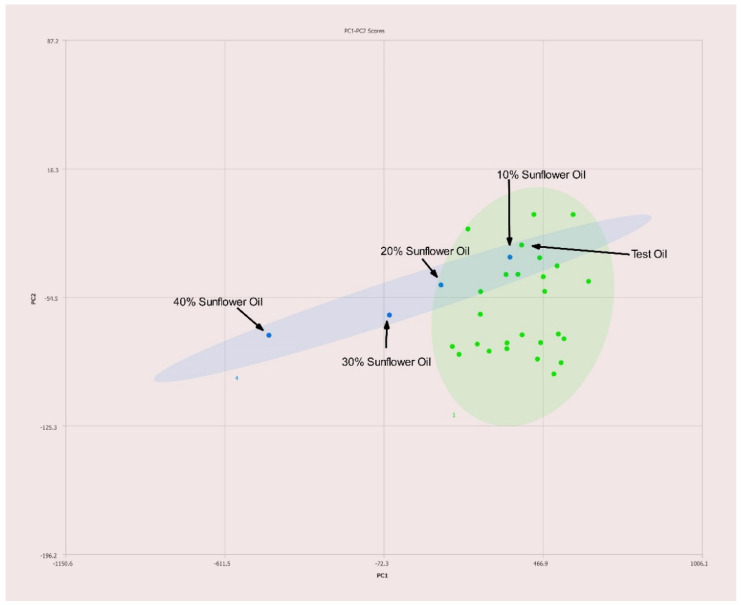
PC1–PC2 plot of 400 MHz 1H NMR spectra of olive oil adulterated with sunflower oil with percentages of adulteration noted. Ellipses represent the 95% confidence interval.

High oleic acid sunflower oil is another ordinarily difficult to detect adulterant of olive oils, yet it is detectable at levels just over 20% *v*/*v* using this method as seen in Figure 10. As the concentration of sunflower oil increases, the sample points on the PCA plot trend toward the pure sunflower oil sample. Differences in the adulterated oil will affect the end result as not all olive oil samples fall exactly together on the PCA plot, but this tool will still identify high-oleic sunflower oil adulterated olive oils at economically viable levels.

**Figure 11:**
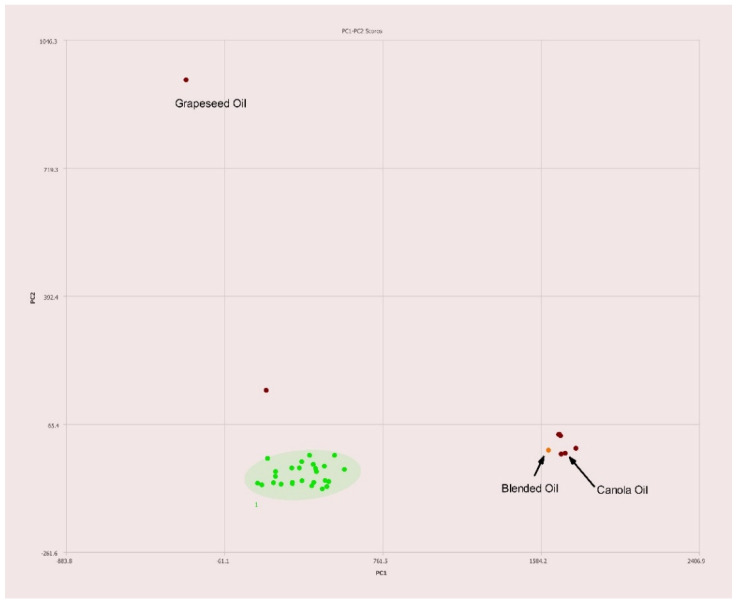
PC1–PC2 plot of 400 MHz ^1^H NMR spectra of a commercially available blended oil composed of 70% canola, 20% grapeseed, and 10% olive oils (*v*/*v*). Ellipses represent the 95% confidence interval.

A commercially available blend of 70% canola, 20% grapeseed, and 10% olive oils was tested for the sake of comparison. As expected, the placement on the PC1–PC2 plot is nearest to canola oil with slight deviation toward both olive and grapeseed oils as seen in Figure 11.

### 3.4. Detection of Adulterated Olive Oils

Upon successfully configuring this method it became clear that two of the olive oils, samples 1 and 27, originally purchased to determine a baseline for genuine olive oils appear to be adulterated themselves (Figure 11) as they lay outside the 95% confidence interval. Sample 1 was sold as a monovarietal arbequina extra virgin olive oil purchased from a from a boutique olive oil shop, and sample 27 was purchased from a grocer specializing in Middle Eastern products and was simply labeled “Moroccan Extra Virgin Olive Oil.” Sample 1 was claimed to have been screened for authenticity via HPLC as part of grading. No testing was claimed for sample 27. Due to placement on the PC1-PC2 plot outside of the 95% confidence ellipse for olive oils, it is likely that both oils are adulterated at relatively low levels. Sample 1 is trending toward peanut or grapeseed oil. Sample 27 is trending toward a cluster of many other oils known to be used as adulterants and as such it is unclear as to which adulterant is included.

## 4. Discussion

Olive oils are known for having low levels of ω-3 fatty acids. By using the expected composition of olive oil during processing of oil spectra it is possible to greatly enhance the adulteration detection capability of a 400 MHz NMR. Typically, NMR spectra is normalized to the tallest peak in each spectrum. However, when one normalizes the intensity of oil spectra to the terminal methyl triplet of ω-3 fatty acids at 0.975 ppm as demonstrated here the spectral differences in non-olive oils are enhanced. As such adulterants such as high-oleic safflower and high-oleic sunflower oils that are ordinarily difficult to detect become readily detectable at economically viable levels of adulteration with a 400 MHz instrument. The data summarized in Figure 9 and Figure 10 demonstrate the enhanced ability to discern olive oils adulterated with high oleic sunflower oil and safflower oil compared to traditional normalization techniques.

Adulteration of olive oils with low ω-3 fatty acid containing oils such as peanut, high-oleic safflower, high-oleic sunflower and high oleic canola oils are still quite easy to detect when using this method. This method does not specifically rely on the presence of sterols, terpines, phenolic compounds, or other low abundance marker compounds that are often destroyed or removed during refining, allowing this method to be used on refined olive oils, as well as virgin, or extra virgin oils.

While effective detection of olive oil adulteration remains a challenge, this method of leveraging processing techniques in order to determine the authenticity of olive oils quickly using more accessible NMR instrumentation should allow for wider availability of NMR-based authenticity testing. Even with only 26 genuine olive oils in this series of experiments, it was possible to detect two probable adulterated olive oils already with real world samples. The accuracy of the model will improve with greater numbers of genuine olive oil samples. The greatest benefit is the ability to screen these oils effectively with a 400 MHz NMR system without the need for a cryogenically cooled probe or specialized diffusion probe [10]. The significantly reduced cost of the equipment needed to perform these analyses and the higher throughput of NMR compared to chromatography-based analyses should make NMR a very competitive instrumentation choice for authenticity analysis purposes.

## Figures and Tables

**Table 1:** List of olive oil samples used.

Sample	Varietal	Grade
1	Arbequina	Extra Virgin
2	Picual	Extra Virgin
3	Nocellara	Extra Virgin
4	Manzanillo	Extra Virgin
5	Hojiblanca	Extra Virgin
6	Coratina	Extra Virgin
7	Koroneiki	Extra Virgin
8	Blend	Extra Virgin
9	Manzanillo	Extra Virgin
10	Hojiblanca	Extra Virgin
11	Blend	Extra Virgin
12	Kilkai	Extra Virgin
13	Manzanillo	Extra Virgin
14	Ascolano	Extra Virgin
15	Arbequina	Extra Virgin
16	None Specified	Extra Virgin
17	None Specified	Extra Virgin
18	None Specified	Extra Virgin
19	Pendolino	Extra Virgin
20	Coratina	Extra Virgin
21	Picual	Extra Virgin
22	Coratina	Extra Virgin
23	None Specified	Olive Oil
24	None Specified	Olive oil
25	None Specified	Refined
26	None Specified	Extra Virgin
27	None Specified	Extra Virgin
28	Blend	Extra Virgin

**Table 2:** Spectral processing parameters.

Parameter	Value
Spectral Reference	TMS
Apodization	0.3 Hz
Phase Adjustment	Automatic
Baseline Correction	Polynomial, order = 5
Intensity Normalization	Peak at 0.975 ppm

**Table 3:** List of blinded regions used in the PCA analysis.

High Frequency Limit (ppm)	Low Frequency Limit (ppm)	Item Eliminated
−3.9	−1.0	Low frequency noise region
−0.2266	0.1892	TMS peak and satellites
6.995	7.006	CDCl_3_ satellite peak
7.195	7.325	CDCl_3_ main peak
7.502	7.548	CDCl_3_ satellite peak
13.02	16.17	High frequency noise region

## Data Availability

Data is contained within the article. The original data presented in this study are available on request.

## Sample Availability

Samples of the compounds are available from the authors.
